# Effect of resistance training plus enriched probiotic supplement on sestrin2, oxidative stress, and mitophagy markers in elderly male Wistar rats

**DOI:** 10.1038/s41598-024-58462-4

**Published:** 2024-04-02

**Authors:** Majid Mohabbat, Hamid Arazi

**Affiliations:** Department of Exercise Physiology, Faculty of Sport Sciences, University of Guilan, P.O. Box: 41635-1438, Rasht, Iran

**Keywords:** Physiology, Environmental sciences, Biomarkers, Health care

## Abstract

This study aimed to determine the effects of resistance training combined with a probiotic supplement enriched with vitamin D and leucine on sestrin2, oxidative stress, antioxidant defense, and mitophagy markers in aged Wistar rats. Thirty-five male rats were randomly assigned to two age groups (old with 18–24 months of age and young with 8–12 weeks of age) and then divided into five groups, including (1) old control (OC: n = 5 + 2 for reserve in all groups), (2) young control (YC: n = 5), (3) old resistance training (OR: n = 5), (4) old resistance training plus supplement (ORS: n = 5), and old supplement group (OS: n = 5). Training groups performed ladder climbing resistance training 3 times per week for 8 weeks. Training intensity was inserted progressively, with values equal to 65, 75, and 85, determining rats' maximal carrying load capacity. Each animal made 5 to 8 climbs in each training session, and the time of each climb was between 12 and 15 s, although the time was not the subject of the evaluation, and the climbing pattern was different in the animals. Old resistance plus supplement and old supplement groups received 1 ml of supplement 5 times per week by oral gavage in addition to standard feeding, 1 to 2 h post training sessions. Forty-eight hours after the end of the training program, 3 ml of blood samples were taken, and all rats were then sacrificed to achieve muscle samples. After 8 weeks of training, total antioxidant capacity and superoxide dismutase activity levels increased in both interventions. A synergistic effect of supplement with resistance training was observed for total antioxidant capacity, superoxide dismutase, and PTEN-induced kinase 1. Sestrin 2 decreased in intervention groups. These results suggest that resistance training plus supplement can boost antioxidant defense and mitophagy while potentially decreasing muscle strength loss.

## Introduction

Aging is a ubiquitous biological process resulting in a progressive and irreversible decline in physical function across all organ systems induced by the accumulation of damage in response to a variety of stressors^1^. The world is facing the problem of an aging population, with approximately 10% of people globally aged 65 or over in 2020. By 2030 and 2050 the proportion of this age group is projected to be 12% and 16%, respectively^2^. Several molecular mechanisms that regulate aging have been discovered, such as telomere dysfunction^3^, loss of proteostasis^4^, mitochondrial dysfunction^5^, stem cell exhaustion^6^, and epigenetic alterations^7^. The aging process is driven by several complex and important pathways, many of which are associated with chronic oxidative stress (OS) caused by elevated levels of reactive oxygen species (ROS)^8^. It has been shown that during aging, the integrated effects of oxidative stress and inflammation largely reduce cellular antioxidant capacity, greatly inhibiting the activation of skeletal muscle satellite cells proliferation and differentiation of myoblasts^9^. However, results in this context are somewhat equivocal since some animal and human studies showed that the activity of muscle antioxidant enzymes included glutathione peroxidase (GPX), glutathione reductase (GR), superoxide dismutase (SOD), catalase (CAT), and glutathione sulfur-transferase were increased significantly with aging in both soleus and deep portion of vastus lateralis muscle^10^. These results were also confirmed by Ji et al. (1990), highlighting that cytosolic (Cu–Zn), mitochondrial (Mn) superoxide dismutase (SOD), and catalase activity increased in comparison with the young group^11^. On the other hand, recent studies suggest that enzymatic antioxidant defense decreases with aging^12^. Also, Kozakiewicz et al. observed that superoxide dismutase (SOD-1), catalase (CAT), and glutathione peroxidase activities were lower in elderly people in comparison with the control group^13^, emphasizing the need for more research in the field.

It is generally accepted that ROS plays a primary role in the aging process, especially in tissues with more pronounced generation of free radicals, such as skeletal muscle^5^. This is a consequence of the high oxygen consumption levels seen in skeletal muscle compared to other tissues, resulting in higher concentrations of ROS^14^. Mitochondria are a major source of ROS in skeletal muscle, and mitochondrial Deoxyribonucleic acid (DNA) may be especially susceptible to oxidative DNA damage^15^. Indeed, intra-mitochondrial ROS generation also exists and is normally generated by tightly regulated enzymes, such as Nitric oxide synthase (NOS) and Nicotinamide adenine dinucleotide phosphate (NAD(P)H) oxidase isoforms^16^, among which NOX 2 and 4 are major sites of ROS during skeletal muscle contraction^17^. Also, xanthine oxidase contributes to this phenomenon. Phospholipase A2 (PLA2) is another known source of intracellular ROS production in muscle. In this pathway, Arachidonic acid, released by PLA2, is a substrate for ROS-generating enzyme systems such as lipoxygenases. Activation of PLA2 can stimulate NOXs, and increased PLA2 activity can promote ROS production in muscle mitochondria and cytosol and release ROS into the extracellular space. Both forms of PLA2 are involved in ROS generation in muscle^18^. Peroxisomes are another site of ROS production in cells. Aberrant ROS generation and oxidative damage have been associated with many aspects of mitochondrial dysfunction in skeletal muscle aging.

It has been well-documented that mitochondrial dysfunction is associated with sarcopenia^1,15,19–21^. Sarcopenia is defined as a decline in muscle mass and strength with age and is now formally recognized as a muscle disease with an International Classification of Diseases, Tenth Revision, and Clinical Modification (ICD-10-CM) diagnosis code^22^. Evidence suggests that OS and decreased antioxidant capacity contribute to this scenario. OS is an imbalance in oxidant and antioxidant levels^23^. Aging has been shown to predispose skeletal muscle to increased levels of OS both at rest and during disuse atrophy^2^, suggesting that OS has a role in mediating disuse-induced and sarcopenia-associated muscle loss^15^. Mitophagy is an autophagosome clearance of dysfunctional mitochondria. Different pathways are identified but the classic and well-known pathway is parkin-dependent mitophagy. Evidence of ubiquitin-independent or PTEN (Phosphatase and TENsin homolog deleted on chromosome 10)-induced kinase 1(PINK)-Parkin-independent mitophagy pathways emerged over the last decade^24–26^. Phosphorylation of stress-sensing protein Sestrin2(Sesn2) by ULK1(Unc-51-like autophagy-activating kinases1) at Ser-73 and Ser-254 acts as the upstream induction signal for autophagic degradation of mitochondria in response to cu-induced ROS. This suggests that Sesn2 is unlikely to be directly involved in the PINK1-Parkin mitophagy mechanism. Loss of phospho-Sesn2 inhibits mitochondria association with autophagosome^27^*.* Studies have shown that depletion of Sesn_2_ in drosophila downregulates AMP-activated protein kinase (AMPK) and upregulates (mammalian/mechanistic target of rapamycin complex 1)mTORC^1^, together leading to the accelerated development of several age-related and obesity-induced pathologies, such as lipid accumulation, mitochondrial dysfunction, and muscle degeneration^28,29^. Impaired mitophagy can lead to dysfunctional mitochondria accumulation with resultant build-up of ROS, ultimately damaging macromolecules in cellular structures. Sesn_2_ enhances autophagic efficiency by recognizing and shipping the damaged mitochondria to lysosomes for degradation^30^. Notably, sestrins expression is specifically high in mouse soleus muscle, which is enriched with oxidative fibers^31^.

Sesn2 is primarily regulated by p53 and acts as a cytoprotective agent against genotoxicity and oxidative stress. The antioxidant function of the Sesns protein was originally attributed to their sulfonic acid reductase activity required for regenerating over-oxidized 2-cys-peroxiredoxins (Prxs) ^32^. Evidence suggests that Sesns, especially Sesn2, can counteract oxidative stress, lessen the mammalian/mechanistic target of rapamycin (mTOR) expression, and promote cell survival^26^. Sesns are thought to attenuate tissue aging through their dual biological activities. Excessive accumulation of ROS and chronic activation of mTORC1 signaling are well-known promoters of tissue aging^26^.

Studies in rodents revealed that Sesn2 expression declines with age in mice^33^. Also, extensive disuse and inactivity have been shown to reduce the level of Sesns expression both in rodents and humans^34^. Sesns were found to be downregulated in frail compared to nonfragile elderly^35^.

Sesn2 was also essential for mediating exercise effects in other organs. Along with this function, it has been reported that inactivation of Sesn2 or Nrf2 (nuclear factor erythroid 2–related factor 2) induced reactive oxygen species-mediated proteasomal inhibition and platelet-derived growth factor receptor beta (Pdgfrb) accumulation. Sesn2 blunts FOXO (Members of the class O of forkhead box transcription factors)-dependent atrogenes in disused muscle. Also, Sesns coaxes autophagy by blunting mTORC1 to protect muscle against aging-associated atrophy^33^.

Recently there has been a huge attention to the application of nutritional supplements to treat adult sarcopenia, with a focus on probiotics, vitamin D, and leucine supplementation (especially lactobacillus and bifidobacterium as the most frequently used forms of probiotics regarding gut-muscle axis to enrich the gut microbiome)^36^. It has been shown that lactobacillus and bifidobacterium can contract sarcopenia both in humans and animals^37,38^, and some studies have suggested its incorporation into the therapeutic process of the elderly. On the other hand, protein supplementation, especially leucine amino acid, attracted great attention and was most often used alone or in combination with resistance training^8^. Another supplement, Vit D3, has been used in different ranges, but the effect of this utilization is controversial and may depend on the level of physical activity of the consumer. These scenarios showed that probiotics' co-administration with protein-contained food (Amino acid-induced leucine) significantly increases maximum concentrations and levels of Amino acids, especially methionine, histidine, valine, leucine, isoleucine, tyrosine^39^, total BCAA^39,40^, and total essential amino acids, which is an important component of sarcopenia therapeutic intervention. On the other hand, prebiotics administration causes an increase in provitamin D3^41^, hence resolving vitamin D deficiency in sarcopenia. Evidence has also highlighted the positive association of the vitamin D/VDR pathway with skeletal muscle mass, function, and regeneration^42^. In addition, the role of vitamin D has been confirmed as a stimulator of PINK1/PARKIN-dependent mitophagy that can control ROS production by deletion of dysfunctional mitochondria^43^. Investigation of the antioxidant action of the mentioned components revealed that vitamin D deficiency was frequently correlated with elevated malondialdehyde (MDA) levels and decreased superoxide dismutase (SOD) activity^44^. Also, it was shown that vitamin D increased the level of TAC and glutathione^45^.

Research on specific probiotics, such as lactobacillus plantarum, has confirmed that co-administration of these probiotics with other substances increases antioxidant activity^46^. Increased TAC, SOD, glutathione peroxidase, and catalase enzyme activity observed with specific pathogen free Kun Ming mice^47^. The antioxidant and anti-inflammatory properties of Bifidobacterium bifidum have been also supported by several studies^48,49^. It seems that antioxidant mechanism of lactic acid bacteria (LAB) is mainly inserted by the ability of LAB in chelated metal ions, autotrophic antioxidant enzyme systems, production of antioxidant metabolites, increased host antioxidant enzyme activity, control of antioxidant signaling pathways, and regulation of intestinal bacteria group^48^. Thus, the administration of these three extraordinary components, their interaction with mitophagy, and antioxidant activity is an interesting scientific issue.

In addition to this attractive contribution of Sesn2 to blunting OS and the somewhat aging process in muscle tissue and other organs and stimulating mitophagy to degrade dysfunctional mitochondria, research on sarcopenia, in which OS and dysfunctional mitochondria accumulate, will yield promising results. This study has focused on the interactive effect of resistance exercise (RE) combined with vitamin D, probiotics, and leucine supplementation on TAC, TOS, SOD, Sestrin-2(Sesn2) and mitophagy markers (PINK and Parkin) in elderly male Wistar rats. Finally, our hypothesis is that exercise may increase mitophagy markers and rise antioxidant activity, but the combination of exercise and supplement may have different impact on sestrin and mitophagy elements which need to evaluate. Likewise, in line with the research evidence, it is expected that both resistance activity and supplementation will improve the antioxidant capacity.

## Results

To homogenize all groups, we used young and old control groups and the young group as the reference for the expected fluctuations in given factors because the research did not follow a pretest–posttest design except for grip strength. Shapiro–Wilk test was used as the normality test in the study variable. The *p*-value of this test is shown in Table 1.

**Table 1:** Shapiro–Wilk normality test in different variables between groups.

Shapiro–Wilk test	OC	YC	OS	OR	ORS
Sesn2					
W	0.9651	0.8848	0.9558	0.9155	0.8737
*p*-value	0.6410	0.3386	0.5954	0.4366	0.3061
Parkin					
W	0.9583	0.9096	0.9104	0.9990	0.9176
*p*-value	0.6073	0.4167	0.4194	0.9400	0.4439
TAC					
W	0.8242	0.8353	0.8710	0.9959	0.9304
*p*-value	0.1736	0.2018	0.2983	0.8776	0.4902
PINK					
W	0.9268	0.7890	0.9542	0.9533	0.9999
*p*-value	0.4766	0.0885	0.5883	0.5841	0.9810
SOD					
W	0.8040	0.8191	0.9452	0.9884	0.9155
*p*-value	0.1242	0.1609	0.5487	0.7939	0.4367
TOS					
W	0.8073	0.8710	0.7695	0.9992	0.9959
*p*-value	0.1321	0.2983	0.0534	0.9475	0.8776

The obtained values indicate the normality of the data distribution among different study variables in all groups. Here, W shows statistics, OC stands for old control, YC for young control, OS for old plus supplement, OR old plus resistance training, and ORS for old plus supplement and resistance training.

### Total oxidant status (TOS)

**Figure 1:**
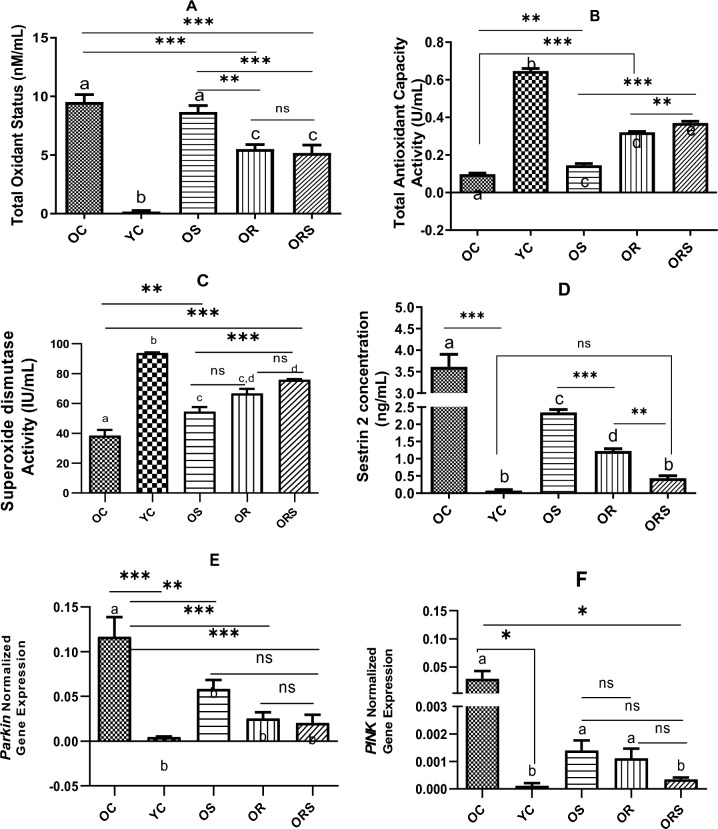
Differences of variables between the intervention and control groups. Graph A shows multiple comparisons between study groups in TOS, B in TAC, C in SOD, D in Sesn_2_, E in Parkin, and F in PINK. OC stands for old control, YC for young control, OS for old plus supplement, OR for old plus resistance training, and ORS for old plus supplement and resistance training. *Denotes significant within-group differences after-training period values (*p* = 0.033), ** (*p* = 0.002), ***(*p* < 0.001), and ns represents non-significant.

After 8 weeks of intervention, Tukey's multiple comparison tests showed significant differences between the YC group with all other groups (*p* = 0.001). However, there were no significant differences between old vs. old plus supplement (OS) groups and old plus resistance (OR) versus old plus resistance training and supplement (ORS) groups. Results revealed significant differences between OC versus OR (*p* = 0.002), OC versus ORS (*p* = 0.001), OS versus OR (*p* = 0.011), and OS versus ORS (*p* = 0.005). TOS was the highest in the OC group, whereas the lowest content was shown in the YC group followed by a decrease in OR, ORS, and OS, respectively (Fig. 1a).

### Total antioxidant capacity (TAC)

The highest TAC was found in YC (*p* = 0.001) and differed from all other groups. TAC was higher in OS (*p* = 0.027), OR, and ORS (*p* = 0.001) compared to the OC group. Significant differences were shown between the OS, OR, and ORS (*p* = 0.001) groups. TAC was higher in the ORS versus OR (*p* = 0.022) group. It seems that resistance training alone and in combination with supplements may have a greater impact on TAC, exerting a synergistic effect on it (Fig. 1b).

### Superoxide dismutase (SOD)

Similar to TAC, SOD activity was the highest in YC compared to other groups (*p* = 0.001). Statistically significant differences were observed between OC versus OS (*p* = 0.011) and OS versus ORS (*p* = 0.001), representing higher values in the resistance training group and resistance plus supplement compared to supplement or resistance alone. There were no differences between the OS versus OR (*p* = 0.052) and OR versus ORS (*p* = 0.175) groups. As shown by the results, resistance training had a major effect on SOD activity compared to supplements (Fig. 1c).

### Sestrin-2 (Sesn2)

Sesn2 level was the highest in OC and lowest in the YC group. Significant differences were shown between YC and all other groups (*p* = 0.001) except for the ORS group (*p* = 0.443). The differences between OR versus ORS (*p* = 0.021) and OS versus OR (*p* = 0.002) were also statistically significant. The results of this factor showed that supplement and resistance training alone and in combination with supplement could reduce Sesn2 activity and propel this to the level observed in the YC group (Fig. 1d).

### Parkin

Parkin normalized gene expression showed significant differences between OC versus YC (*p* = 0.001), OS (*p* = 0.039), OR (*p* = 0.002), and ORS (*p* = 0.001) groups after a period of training. No differences were observed between other groups. Parkin activity tended to decrease with the application of exercise and supplements but was the highest in the OC group (Fig. 1e).

### PTEN-induced kinase (PINK)

Analysis of gene expression indicated that PINK expression level was different between OC versus YC (*p* = 0.045) and OC versus ORS (*p* = 0.046), groups but no statistical differences were found between other groups. As revealed, PINK expression was reduced to the level of the young control group with exert intervention (exercise and supplement) and a combination of both interventions (Fig. 1f).

### Grip strength

**Figure 2:**
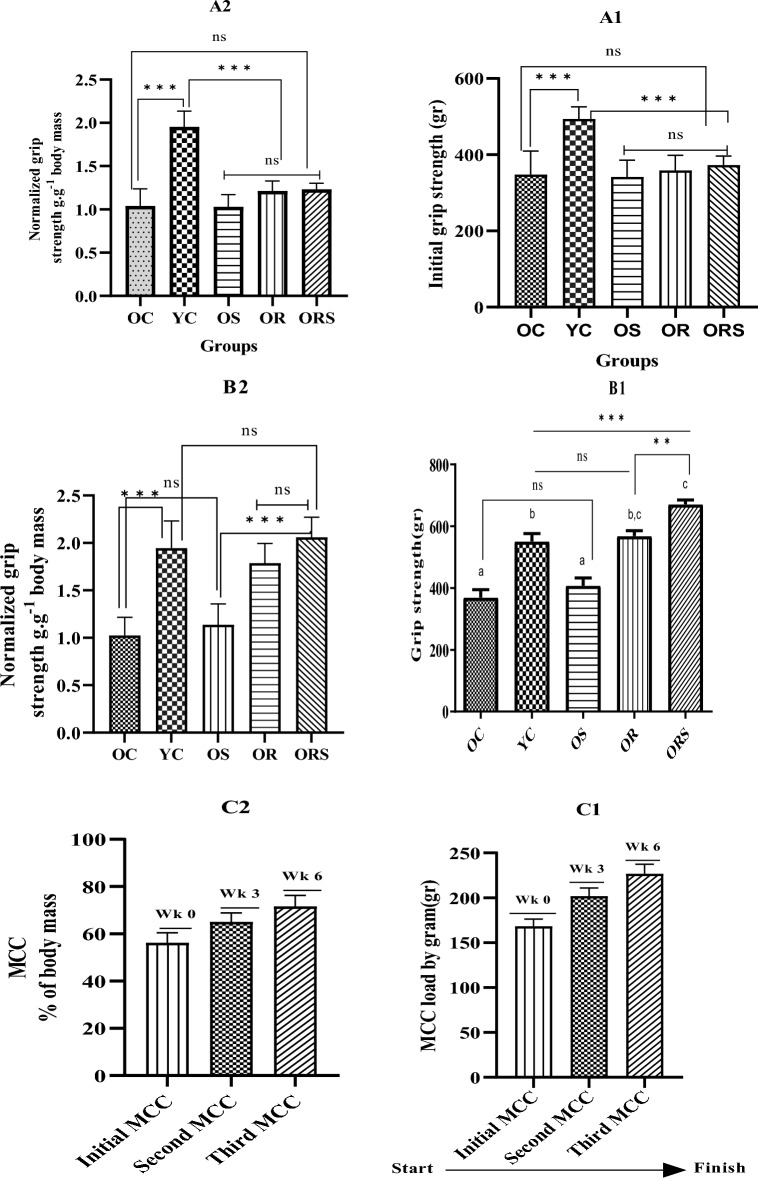
Grip strength in the absolute and relative values before and after the intervention and maximum carrying capacity in three stages among the training groups. OC stands for old control, YC for young control, OS for old plus supplement, OR for old plus resistance training, and ORS for old plus supplement and resistance training. Wk0 shows the baseline of MCC, Wk3 shows MCC at the end of the 3rd week, and Wk6 shows MCC at the end of the 6th week. A1 and A2 show the baseline of the grip strength before starting the protocol, B1 and B2 indicate post-intervention grip strength and C1 and C2 represent MCC in three stages, both in relative and absolute values, respectively. *Denotes significant within-group differences between after-training period values (*p* = 0.033),** (*p* = 0.002), ***(*p* < 0.001), and ns stands for non-significance.

Our findings showed no differences between the OR vs. YC (*p* = 0.980) groups. Besides, supplement alone did not impact the grip strength (OC vs. OS; *p* = 0.740) but had more effect on grip strength in combination with resistance training (ORS vs. OR; *p* = 0.020) and (ORS vs. YC; *p* = 0.005), revealing a synergistic effect on this parameter (Fig. 2).

### Animal drop number (death) during study

There was no drop in the number of animals in the study groups at any stage of the study.

**Figure 3:**
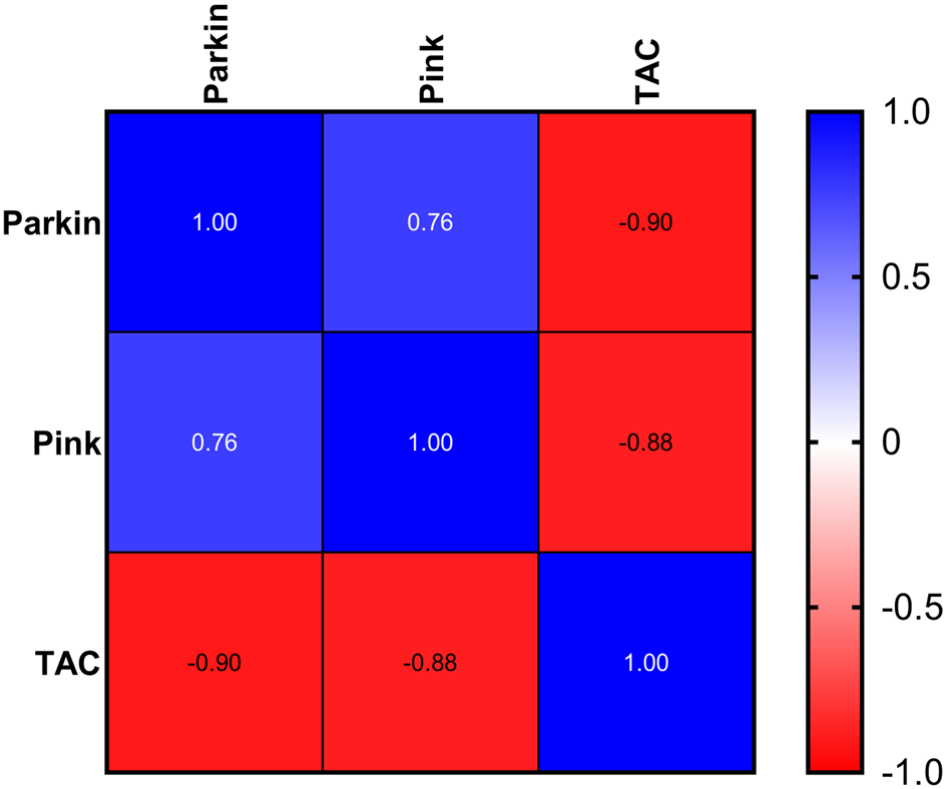
Heatmap shows the correlation between Parkin, PINK, and TAC. Significance was set on (*p* ≤ 0.05). TAC and PINK stand for total antioxidant capacity and PTEN-Induced Kinase1, respectively.

### Correlation

**Figure 4:**
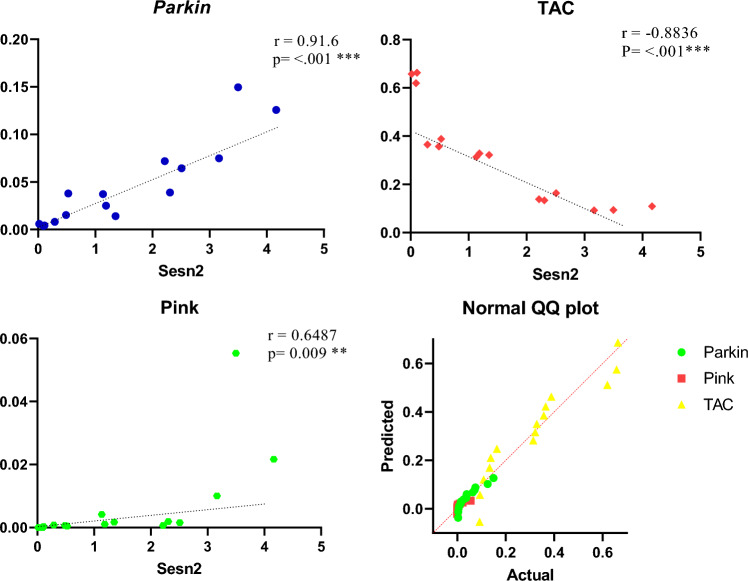
Pearson correlation test between Sesn2 and Parkin, A; Sesn2 and TAC, B; Sesn2 and PINK, C; and Normality test, D. TAC stands for total antioxidant capacity, PINK for PTEN-Induced Kinase 1, and Sesn2 for sestrin2. The mean line and *p*-value were also reported in each panel of figure. *Denotes significant within-group differences between after-training period values (*p* = 0.033),** (*p* = 0.002), ***(*p* < 0.001), and ns stands for non-significant.

The correlation between Parkin and PINK, Parkin and TAC, and PINK and TAC were assessed in y and y pair and reported with a heatmap correlation matrix (Fig. 3). Results showed a strong correlation between Sesn2 vs. Parkin (r = 0.91.6; *p* < 0. 001) (CI 95% (0.7465 to 0.9703), Sesn_2_ vs. PINK (r = 0.6487; *p* < 0.009 (CI 95%, 0.2044 to 0.8714), and strong inverse correlation between Sesn2 and TAC (r = − 0.8836; *p* < 0. 001(CI 95%, − 0.9609 to − 0.6783) (Fig. 4).

## Discussion

The present study aimed to assess and compare the effects of resistance training and supplement after an 8-week program on mitophagy, redox balance indices (i.e., parkin and PINK, total oxidant status, total antioxidant capacity, superoxide dismutase, and sestrin2), and functional capacity (grip strength) in young vs. old male Wistar rats. Our study suggests that both training and supplementation interventions might be effective in improving TAC and SOD activity which were aligned with our hypothesis. But on the contrary, Sesn2, parkin, and PINK activities decreased; however, the YC group demonstrated significantly better characteristics toward optimal status in all factors (i.e., TAC, and SOD activities). In contrast, the OC group demonstrated significantly lower characteristics in all factors among other groups during 8 weeks of protocol (i.e., higher TOS, lower TAC, and SOD activities), which is in line with our study hypothesis.

Sesn2 activity was the lowest in the young control group compared to all groups, except for the ORS group, which showed no differences in Sesn2 after 8 weeks of intervention (resistance training along with supplementation). Resistance training alone had a greater effect on Sesn_2_ decrement than the supplement group, but resistance training had a synergistic effect on this drop in Sesn2 activity. As revealed, both exercise and supplementation could decrease Sesn2 levels and normalized these levels for the aging group. One of the main actions of Sesn2 is its antioxidant capacity and contract with ROS accumulation^50^. Literature on the Gut-Muscle axis has generally accepted that the application of probiotics can contract microbiome changes that drive inflammation and ROS production by pathobionts^51^. In the process of searching for the effect of resistance training on the Sesn2 level, we found out that most interventions in research works have focused on aerobic exercise, and few studies have used resistance training modality. Research on the effect of exercise on sestrin protein expression in humans and rodents shows a lack of consistency; for instance, Zeng et al. reported that chronic resistance training did not affect Sesn2 levels in humans ^52^. Contrary to this finding, Correˆa et al. reported that the Sesn2 level increased after six months of resistance training in dialytic patients^53^. However, these differences may depend on population variation between these studies. Several in vivo and in vitro studies have indicated that lactobacillus and bifidobacterium strains have great antioxidant capacity. It seems that these probiotics work through various factors such as, the synthesis of non-enzymatic antioxidants and metabolites with antioxidant properties^54,55^. Wang et al. showed that supplementation with bifidobacterium longum and lactobacillus plantarum strains insert strong antioxidant features in A7R5 Cells^56^. This may be due to the effect of these strains on the amount of reactive oxygen species, which has been confirmed through the reduction of sestrin production and also the reduction of mitophagy activation. However, it should be kept in mind that the probiotic strains used in various studies were somewhat different from the present study.

According to our finding, a decrement in the serum level of Sesn2 can be attributed to leucine administration because Sesn2 has been defined as a putative leucine sensor in HEK-293 T cells via the inhibition of the GATOR2 subcomplex^57^. and selectively promotes dephosphorylation of Sesn2, causing it to dissociate from and thereby activate GATOR2, leading to the activation of mTORC1^58^.On the other side, TOS was higher in the OC group and didn’t change by the supplement administration, thereby minimally changing when applied simultaneously with resistance training and revealing the larger blunting effect. Resistance training positively affected the TOS decrease when compared with supplement.

TAC is the body's ability to contract with excess free radical accumulation and maintain redox balance. Eight weeks of supplementation and resistance training increased the activity level of TAC, but both resistance and resistance plus supplement couldn’t increase this level of activity to those seen in the YC group. Our finding showed the reinforcing effect of exercise on supplement in the context of antioxidant capacity, but resistance training was more effective than supplementation alone. Consistent with this result, Mesquita et al. stated that six weeks of resistance training increased total antioxidant capacity in older adults^59^. Another study by Amiri et al. proved that 10 weeks of creatinine supplementation enhanced antioxidant capacity by inserting a synergistic effect on resistance training^60^. Overall, this finding suggests that resistance training could ameliorate oxidative stress by increasing TAC, and it did not depend on the type of resistance training and training intensity, although it changed by 4% in intense training^61^. In a meta-analysis of 11 randomized clinical trials, Morvaridzadeh et al. reported that pomegranate juice had no incremental effect on TAC nor on decreasing MDA^62^. Another study by Atashak et al. showed that ginger supplementation accompanied by resistance training for 10 weeks increased TAC in obese men^63^, which is consistence with our finding. As found by the researcher, only a few studies investigated the supplement used in the present study, which was a combination of probiotics enriched with vitamin D and leucine amino acids. Jamilian et al. investigated the effect of probiotic supplements, along with vitamin D, on women and reported that this supplement reduced MDA and increased total antioxidant capacity^64^. This result was confirmed by Abboud^65^ and Ostadmohammadi^66^.

This finding illustrates that both exercise and supplement can improve the enzymatic antioxidant system. Another study confirmed that different resistance-type training had a positive effect on SOD^67^. In line with this finding, Alikhani et al. reported that long-term resistance training increased TAC both in young and older trained women while also revealing that resistance training could act similarly in older and younger subjects in promoting antioxidant capacity^68^.

SOD is responsible for the dismutation of O_2_ radicals into H_2_O_2_^63,69^, which is in alignment with the previous literature^67,70^. In contrast with our finding, Stefani et al. assessed the effect of 8 weeks of resistance training by 65% to 75% of 1MR in rats and showed that SOD activity was lower in the trained and supplement group, which could be attributed to the tissue sampling of the mentioned study^71^. Silva et al. examined the taurine supplementation following eccentric exercise on oxidative stress and indicates that SOD activity didn’t change. The inconsistency between our results may depend on the difference in intervention because they investigated one session and the acute effects. Long-term resistance training did not affect the protein content of both CuZnSOD, and MnSOD^72^, revealing that exercise effect may depend on some factors such as age and gender of participants, exercise intensity, type of supplement, and intervention duration. Generally, it is worth noting that exercise alone has a more effective impact on antioxidant enzyme activity than supplementation. Also, it has been reported that polyphenol-containing plant-based foods may contract with exercise to increase the antioxidant effect of exercise when co-administrated^67^.

As previously mentioned, Sesn2 correlates with decreased oxidative stress and can inhibit mitochondrial dysfunction, highlighting the importance of investigating the relationship between the well-known mitophagy pathway (PINK and Parkin) and Sesn2 activity levels. In this regard, our finding suggests that the administration of supplement, along with resistance training, decreases parkin and pink activity to a level similar to what is seen in the young control group. Despite endurance training, few studies have investigated the impact of resistance training on mitophagy to date, and most of them are equivocal. For instance, Ogborn et al. showed that PINK and Parkin mRNA expression did not change after a session of unaccustomed resistance exercise^73^. By contrast, as earlier discussed, Sesn2 could be phosphorylated on Ser-73 Ser-254 residues of Sesn2 by ULK-1 and reduced p62 activation^27^, boosting autophagic degradation of mitochondria and possibly increasing PINK and parkin activity, although it has not been directly tested. It was also confirmed that probiotics increased activity of PINK and Parkin to blunt the further increase in ROS^74^. vitamin D can suppress mitophagy by inhibiting mitochondrial fission proteins, phosphorylated dynein-related protein 1 (pDrp1), and mitochondrial fission factor (Mff)^75^. Consistent with our result, Castro et al. indicated that Parkin protein level decreased after a session of resistance training^76^. One of the outstanding results of our study is the inverse relationship between antioxidant enzyme (TAC) with parkin and PINK. We postulated that probiotic supplementation would blunt ROS accumulation, thus reducing TAC in response to exercise. On the other hand, activity level of TAC decreased when parkin and PINK mRNA expressions were high (activating mitophagy to neutralize dysfunctional mitochondria and reduce the subsequent ROS production and accumulation). In support of this finding, Palacino et al. revealed that the level of proteins involved in protection from OS decreased in parkin-deficient Mice^77^. This finding was also confirmed by Hyun et al., who demonstrated that overexpression of parkin was associated with reduced antioxidant defense, although this marker (protein carbonyls, 3-nitrotyrosine-containing proteins) was different from our study^78^. Thus, it could be concluded that increases in parkin and PINK mRNA expression would decrease TAC by blunting ROS accumulation through the deletion of dysfunctional mitochondria by recruiting mitophagy since the deficiency of parkin or PINK increases oxidative stress due to the accumulation of dysfunctional mitochondria^79,80^. Based on the current finding, it can be postulated that an increase in Sesn2 is accompanied by parkin and PINK mRNA expression, as indicated earlier. Other research in agreement with our findings reported that Sesn2 mediated cytosolic interaction of parkin and beclin1^81,82^. Finally, our finding indicates a drastic increase in grip strength in the co-administration of resistance training and supplements. Vitamin D can bind to VDR and induce muscle hypertrophy by phosphorylating mTOR and their downstream target^83^. This finding was consistence with the Gkekas study, which examined the effect of vitamin D and protein supplementation on strength and muscle mass in sarcopenia, indicating increased grip strength but no effect on muscle mass and muscle cross-sectional area^84^. This result was confirmed by a later finding by Yang et al., who reported the effect of vitamin D on strength and level of physical activity^85^. In a meta-analysis of randomized controlled trials, Prokopidis et al. reported that probiotic supplementation increased both muscle mass and overall muscle strength^86^. In the study of Rondanelli et al., probiotics, leucine, and omega-3 fatty acid were used as potential nutritional supplements in the treatment of sarcopenia, which were largely similar to the present study in terms of constituents. The results of the study, consistent with the findings of the present study, showed an increase in the muscle mass of the limbs, grip strength, and also a significant decrease in visceral fat^40^. In addition, in our study, the intervention groups of exercise and nutrition showed a small amount of weight gain or weight change compared to other groups. This result can be attributed to the effect of leucine and probiotics on the enrichment of intestinal contents and reducing harmful metabolites and fat, which is also confirmed by Celik et al. and Tarakci et al.^87,88^. Although its mechanism of action is poorly understood, it may be attributed to the effect of reducing the calorie intake and inflammation caused by probiotics, diminishing the percentage of fat due to improved cell sensitivity to insulin, and increasing fat metabolism and the percentage of muscle due to leucine. It should be noted that our study was performed on rodents and in combination with probiotic and leucine amino acids, necessitating careful consideration when interpreted by other human research and those conducted only on supplement results.

Finally, in our knowledge of sestrins role in antioxidant capacity and faciliatory role in mitophagy, it is concluded that sestrin increases mitophagy and prevents the accumulation of ROS through the reduction of dysfunctional mitochondria, possibly eliminating the need to increase antioxidant defense. On the other hand, different components of our supplement acted separately on different sites and had synergistic effects on each other. For instance, vitamin D, leucine^89^, and probiotics all have antioxidant role, confirmed based on studies and scientific evidence, while simultaneously indicating a stimulating effect to increase muscle mass through improving intestinal content, regulating and maintaining the integrity of epithelial barriers against pathogenic agents, and improving absorption. However, the interactive effect of different components of the supplement has not been investigated in similar study, highlighting the need for more studies in this field. Overall, we suggest that this supplement in combination with resistance training would be a feasible tool to apply to the treatment regime of sarcopenia and other age-related diseases with oxidative stress and mitochondrial dysfunction as their underlying cause. In addition to the concepts alluded above, it should be noted that the small number of samples as a constraint in the present study may be an interfering factor in the logical conclusion of the findings. Also, among comprehensive profile of antioxidant markers and enzymes, we just assessed mentioned enzymes that may impose some limitation to spotlight on our study finding. As a conclusion, our study showed that exercise and supplementation and their combination, despite the improvement of the antioxidant profile and reduction of oxidant levels, did not lead to an increase in mitophagy, contrary to what we thought, and also reduced the amount of sestrin2 as a mitophagy stimulator, which, of course, considering the other role It (antioxidant activity) seems logical. It was also seen in this study that exercise alone has a greater effect on mitophagy and sestrin factors, although these effects were not significant. In the end, it should be noted that due to the low sample size, the results should be interpreted with caution.

## Methods

### Study design

At the first step, study design was approved by the scientific review board of the University of Guilan. Then, the research protocol received approval from the animal research ethics committee of Shahid Beheshti University (IR.SBU.REC.1402.052), indicating that it was conducted under the ARRIVE and PREPARE guidelines and the 3Rs principles. This study examined the effects of resistance training (RT) with or without supplementation during 8 weeks in aged male Wistar rats. The study was conducted in an experimental design performed in a laboratory setting. Initially, rats were subjected to a number-matched group and performed the same training program (3 days per week, all using the same protocol) in training group and training plus supplement group accompanied by a supplementation (5 days per week) that differed in the control and the training alone. After training protocol, some groups had oral gavage of supplementation. During the period of training, rats regularly suffered weight control weekly. Two days after the end of the training regime, grip strength was assessed, and all animals were put in a Co_2_ chamber for safe and painless anesthesia, after which they were sacrificed to collect the following data: weight, blood sample (3mililiter), and soleus muscle tissue. Collected samples were used to analyze with immunoassay and PCR-Real time techniques.

### Participants

Thirty-five (5 per group) male Wistar rats plus 2 additional rats per each group for reserve in two age groups(Young 8–12 weeks and 18–24 months old)) were brought from RAZI institute to engage in this study at the start of the experiment and were randomly separated into five experimental groups, including an old control group that continued to normal life cycle (OC; n = 5), a young control group that was in active as same as the old control group (YC; n = 3), old resistance training group (OR; n = 5) that was trained using ladder climbing protocol, old resistance training plus supplement group (ORS; n = 5), and old supplement group (OS; n = 5) that used the supplement and was untrained. We used the resource equation approach to calculate the sample size of the study, which is as follows:

For one-way ANOVA, the between-subject error DF (the within-subject DF) is calculated as: DF = *N* − *k* = *kn* − *k* = *k*(*n* − 1), where *N* = total number of subjects, *k* = number of groups, and *n* = number of subjects per group. By rearranging the formula, *n* is given as:

$$n = \, DF/k + \, 1$$

Based on the acceptable range of the DF, the DFs in the formulas are replaced with the minimum (10) and maximum (20) DFs to obtain the minimum and maximum numbers of animals per group: minimum n = 10/k + 1 and, maximum n = 20/k + 1, which result in a range of 3 to 5 sample per group in our study^90^.

### Procedures

#### Animal housing

**Figure 5:**
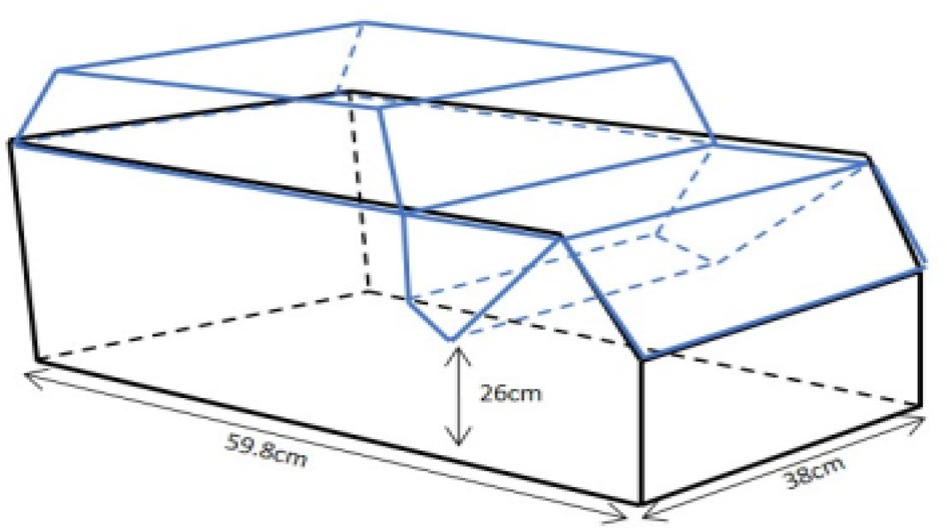
Cage used to accommodate rats ^93^.

At the start of the experiment, rats were placed in collective home cages with members of the same group (five animals per cage with 2 other animals in another cage for reserve) with water and standard rodent chow (ingredients consisting of Carbohydrate 48.8 gr, Protein 21gr, Fat 3 gr, Calcium 0.8 gr, Phosphorus 0.4 gr, Fiber 5 gr, Moisture 13%, Ash 8 gr and Total energy of 306.2 per 100 g of chow (kcal/100 g))^91^ offered ad libitum, and exposed to 12 h light/dark cycles with a temperature of 20–22 °C. The mean weight of our animals was 283.6 ± 8.79 in YC and 342.17 ± 26.65 in old groups. In addition, we used rodent rooms, which had direct-exhaust IVC systems and air ventilation at rates of 5 to 6 air changes per hour (ACH). We housed animals in standard laboratory situations, where a large Euro standard type IV cage was used as shown in Fig. 5.^92^

#### Protocol

For familiarization, rats were adapted to an RT protocol of climbing a vertical ladder (1.1 m; 0.18 m, 2-cm grid, 80º incline) for three non-consecutive days (48-h rest intervals) without any overload. The load apparatus (weight clip) was fixed to the tail by wrapping the proximal portion with a self-adhesive foam strip (for which we used a piece of glue). *The weight value was obtained (as a percentage, for example, 75 at the time of 75%) by dividing the weight of the rat by 1000 and multiplying it by the desired weight number.* The rats were placed at the bottom of the ladder and familiarized with climbing. Although all rats didn’t respond similarly to familiarization or adaptation period, three days was enough to get the desired number for starting protocol (n = 9 three in each experimental group).Two days after the initial familiarization, a new opportunity arose in the meeting to determine the maximum carrying capacity to get acquired rats that were not well adapted. We don’t use pinching to stimulate rats to initiate climbing and rats voluntarily started climbing and we just insert pulling for assistance. In a day after familiarization, rats that did not climb or needed more stimulus to start and maintain climbing were removed from the study. Some rats had a jumping pattern to climb, while some other just climbed step by step, which wasn’t the criteria for our study.

The rats rested for 2 min at the top of the ladder. This procedure was repeated until they would voluntarily climb the ladder for three consecutive turns without any stimulus. To assess the maximum carrying capacity (MCC) two days after the familiarization, each rat was evaluated to determine its maximum carrying load, which consisted of 4–9 ladder climbs with progressively heavier loads. The initial climb was performed with 75% of the animal’s body weight. Upon successful completion of this load, an additional 30 g weight was added to the load clip. The highest load that the animal successfully carried through the entire length of the ladder was considered the maximal carrying capacity for that training session. Failure was determined when the animal could not progress up the ladder after three successive stimuli to the tail. RT was conducted 3 times per week (Saturdays, Mondays, and Wednesdays) for 8 weeks. Ladder climbing was permitted to complete 8–12 dynamic movements in each climbing. The climbs comprised carrying progressive loads of 65, 75, 85, % of the maximum carrying capacity of each rat. RT sessions consisted of 5–8 movements per climb. In total, each rat performed 7 whole ladder climbing. Each session approximately lasted for about 15 min for each rat. It is noteworthy that the mentioned reference was considered as the target point regarding the training intensity. However, the training intensity was adjusted according to the ability of the rats to carry the load, as shown in C part of Fig. 2. The range of intensity was equal to 50–64% in the first three weeks, 59–72% in the second three weeks, and 65–83% in the last two weeks and was rounded to upper domain. The resting period between each climb was 2 min. The RT protocol was adapted from de Farias Junior, et al.^92^ but modified to 8 weeks. It should be noted that even at first session of training or MCC estimation session, rats couldn’t carry 75% of their body weight, and we had to decreased load to the level that they could carry it for successful climbing. Also, in the final session, none of experimental groups could carry 100% of their body weight, and therefore, no 30 g was added. Addition of weight or progression from one load to another load was done in each two to three weeks. Training sessions were conducted in the morning between 10–12 A.M.

### Supplementation ingredients and process

#### Supplement composition, preparation, storage, and admistration

Lactiplantibacillus plantarum (LP)(PTCC: 1058) and Bifidobacterium bifidum (PTCC:1644) were purchased from the Iranian Research Organization for Science and Technology (IROST). The bacteria were cultivated in a MRS agar medium (Merck, Germany) at 37 °C for 24 h. Before supplementation, cells were centrifuged at 3000×*g* for 10 min and washed twice with phosphate-buffered saline (PBS). Pellets were resuspended in PBS, pH 7.2^94^.

The human dose of Lactobacillus plantarum (LP), 2 × 10^10^ CFU per day, which was a low dose selected by Lee et al.^95^, was modified in our study.

The rat LP dose (2.06 × 10^8^ CFU/kg) was converted from a human equivalent dose (HED) based on body surface area according to the following formula from the US Food and Drug Administration: assuming a human weight of 60 kg, the HED for 2 × 10^10^ colony-forming units (CFU) ÷ 60 (kg) = 33.33 × 10^7^ × 6.2 = a rat dose of 2.06 × 10^8^ CFU/kg;^95^ the conversion coefficient 6.2 was used to account for differences in body surface area between a rat and a human, as previously described by Nair et al.^96^.

Bifidobacterium bifidum (PTCC:1644) was obtained from the IROST. To activate the bacteria according to the manufacturer's suggestion, the probiotic was inoculated in Erlenmeyer flask containing 100 ml of peptone water and the resulting media were kept in a greenhouse at 37 °C for 24 h. Then, to form colonies, the contents of Erlenmeyer flask containing activated probiotic were cultured in a plate containing MRS agar under anaerobic conditions (Surface plate method) and kept in a greenhouse at 37 °C for 24–48 h. For Bifidobacterium bifidum, we used dose of 5 × 10^6^ CFU/rat/day according to Khailova et al.^97^.

Vitamin D (Sun Vit®, Iran Hormone) was purchased (Milad Poya company) in the form of ampoules form and mixed with sesame oil (621.7 IU = 0.94 mg vitamin D + 1 ml sesame oil)^42^. Then mixed with suspension of L-leucine that was prepared based on rat volumes corresponding to 0.135 g L-leucine/kg body wt^98^. Finally, probiotics were added to these ingredients, as previously explained, and used by oral gavage.

The supplement was stored at a temperature of 4 °C in a normal refrigerator. Then it was taken out of the refrigerator ten minutes before use and removed from the vial using a syringe, and gavage was performed through the mouth using 16–18 ga stainless steel Feeding sterilized Tube.

#### Grip strength

A modified forelimb grip strength test was used to evaluate grip strength. The tail of a rodent that grips a bar connected to a monitoring device is pulled horizontally by the examiner and the maximal value is recorded as the forelimb grip strength in three trials ^58^.

#### Blood sampling

Three milliliters of blood were obtained by cardiac puncture into sterile vacuum tubes with anticoagulant (EDTA). The collected blood samples were stored at refrigerator (4 °C) for 5 days. Then, samples were taken out and plasma was separated by centrifuge based on the manufacturer's protocol for each variable, which was set at 1000×*g* (or 3000 rpm) in our study for approximately 20 min and used to assess factors. Indeed, to ensure quality blood sample collection, we collected samples in standard laboratory setting, where all devices were calibrated and all staff were trained.

### Soleus muscle extraction and sampling

For this purpose, we first shaved the rats’ hindlimbs on the sample leg. A plexiglass platform was then utilized to fix the rats for surgery. The first incision was through the skin from the heel to the vertebral column. We used scissors to separate the skin from the underlying tissues, after which the superficial muscle layer, which was the gastrocnemius muscle, was cut and removed. Then soleus muscle was cut distally from its insertion and proximally from medial and lateral gastrocnemius muscle heads, completely taken, and immediately stored at a temperature of -80 c in the freezer for 2 weeks. The samples were then extracted from their storage and proceeded to subsequent processes for RNA purity and extraction as below.

#### Gene expression

*RNA extraction from muscle tissue sample*: The soleus muscle from all rats of each group (n = 3/group) was completely taken, and 50 mg of this was used for the analysis. Initially, the samples were macerated by hand using liquid nitrogen with a pistol and homogenized in TRIZOL reagent (Invitrogen Corporation, California, USA) with a PowerGen 125 homogenizer (Fisher Scientific, Pittsburgh, USA). Subsequently, total RNA was extracted according to the TRIZOL manufacturer’s protocol. The RNA pellet was resuspended in nuclease-free water and stored at − 80 °C. The Nanodrop 2000 (Thermo Scientific, Wilmington, USA) was used to measure the RNA concentration and purity (ratios 260/280 nm and 260/230 nm). The purity of the extracted RNA samples was evaluated by checking the OD ratios obtained in conventional spectroscopy. The optical absorption ratio of 260 nm to 280 nm (260/280 ratio) was between 1.9 and 2.0 due to lack of access to bioanalyzer for the RNA integrity number (RIN) calculation.

#### qRT-PCR Reverse Transcription

Before reverse transcription, total RNA (1 μg) from each sample was treated with deoxyribonuclease I (DNase I; Invitrogen Corporation, California, USA) according to the manufacturer’s instructions to remove genomic DNA. Subsequently, treated RNA was reverse-transcribed into cDNA with MMLV reverse transcriptase (Promega Corporation, Madison, USA) and oligo (dT) (Promega Corporation, Madison, USA). After the reaction, the samples were stored at − 20 °C in a freezer. cDNA was prepared from 15 Rats 3 in each group. Quantification expression level of the desired gene was calculated by the formula 2^−ΔΔCT^ in the following way. First, the threshold cycle of the desired gene of each sample was subtracted from the threshold cycle of the housekeeping gene of the same sample (∆Ct = Ct Target-Ct Housekeeping) In the next step, we subtracted the ∆Ct of each sample from the sample that needed to be compared and multiplied the negative number obtained to the power of two and obtain the relative expression of PINK and Parkin genes.

The sequences of the primers used are reported bellow.

r-PINK: F: AATAGTGTGGGTCTCAGCCTTG

R: TATCAGTGTCCCGTTACCTCCC

R- Parkin: F: GAACTGTGGCTGTGAGTGGA

R: CAAAGGTAGGAGTCTGAGGTGT

r-GAPDH: F: AGGTCGGTGTGAACGGATTTG (housekeeping gene)

R: TGTAGACCATGTAGTTGAGGTCA

Three independent RNA extractions were prepared for each sample. Differences between genotypes were assessed by ANOVA.

A blood sample was taken for SOD, TAC, and TOS and used for assessment with enzyme-linked immune sorbent assay (ELISA) method based on the manufacturer's protocol. The assessment was done using a rat SOD kit (Brand ZellBio GmbH (Germany), CAT NO: ZB SOD), TAC kit (Brand ZellBio GmbH (Germany), CAT NO. ZB-TAC), TOS kit (Brand ZellBio GmbH (Germany), CAT NO. ktos-96) and Sestrin 2 kit(Brand myBiosource kit, CAT NO. MBS058466) and expressed as IU/mL, U/mL, nM/ml, and ng/mL, respectively.

#### Data analysis

All values have been presented as mean ± standard deviation (SD). Data analyses were conducted using the Statistical Package for the Social Sciences (SPSS version 27.0 Chicago, IL). The normal distribution of data was tested by the Shapiro–Wilk test. The one-way ANOVA was used to determine the differences between groups when a significant F value was achieved (Supplementary file), and multiple comparison Tukey post hoc tests were performed to identify the differences between the means within groups. The level of significance was set at *p* ≤ 0.05. To determine the relation between dependent variables, the Pearson parametric test was used. GraphPad Prism (Version 9.5.1) was applied to illustrate graphs and some statistical analyses.

### Supplementary Information

Supplementary Information.

## Data Availability

The supporting data presented in this study and its supplementary information are available at https://doi.org/10.5281/zenodo.10116266.
